# Remote Patient Education for People Living with an Ostomy: An Italian Expert Consensus Using a Modified Delphi Method

**DOI:** 10.3390/nursrep16060203

**Published:** 2026-06-15

**Authors:** Giulia Villa, Andrea Poliani, Alessia Campoli, Annarita Coppola, Francesco Carlo Denti, Rossella Guzzi, Danila Maculotti, Marina Perrotta, Clara Salazar, Giovanni Sarritzu, Monica Sgherri, Antonio Valenti, Pier Raffaele Spena, Duilio Fiorenzo Manara

**Affiliations:** 1 Center for Nursing Research and Innovation, Faculty of Medicine and Surgery, Vita-Salute San Raffaele University, 20132 Milan, Italy; villa.giulia@hsr.it (G.V.); manara.duilio@hsr.it (D.F.M.); 2 Department of Biomedicine and Prevention, Faculty of Medicine, University of Rome “Tor Vergata”, 00133 Rome, Italy; alessia.campoli@ifo.it; 3 Nursing Research Unit IFO, IRCCS Regina Elena National Cancer Institute, 00144 Rome, Italy; 4 Surgery Department Head Nurse, S. Anna and SS. Madonna della Neve, Boscotrecase, ASL Napoli 3 Sud, 80042 Napoli, Italy; annarita.coppola1983@gmail.com; 5 Stoma Care Unit, San Raffaele Scientific Institute, 20132 Milan, Italy; denti.francescocarlo@hsr.it; 6 Italian National Association for Incontinence and Ostomy Patients, Federazione delle Associazioni Incontinenti e Stomizzati (FAIS), 20133 Milano, Italy; r.guzzi@faisitalia.it (R.G.); m.perrotta@faisitalia.it (M.P.); c.salazar@faisitalia.it (C.S.); m.sgherri@faisitalia.it (M.S.); p.spena@faisitalia.it (P.R.S.); 7 Fondazione Poliambulanza Istituto Ospedaliero, 25124 Brescia, Italy; macudani71@gmail.com; 8 Stoma Care Unit, Azienda Ospedaliera Universitaria di Cagliari, 09123 Cagliari, Italy; g.sarritzu@libero.it; 9 Stoma Care Unit, Mauriziano Hospital, 10128 Turin, Italy; antonio2valenti@gmail.com

**Keywords:** consensus, Delphi technique, education, ostomy, remote education, nursing, surgical stomas

## Abstract

**Introduction:** Remote education is increasingly used in ostomy care, but its components, timing, governance, and evaluation remain inconsistently defined. This study aimed to develop practice-oriented recommendations for implementing remote patient education for people living with an ostomy. **Methods:** An Italian expert consensus using a modified Delphi method and reported according to the ACCORD guidelines was conducted. An expert panel (n = 11), recruited nationally, included stomatherapists (n = 6) and people living with an ostomy (n = 5). Round 1 comprised a remotely conducted focus group to generate and refine statements informed by a targeted literature search. Rounds 2 and 3 were anonymous online surveys in which panelists rated statements on a four-point Likert scale and could provide comments or propose additional items. Consensus was predefined as ≥75% agreement. **Results:** Response rates were 100% across the three rounds (October–November 2025). The panel achieved consensus on 8 definitions and 14 statements, organized into six domains: (1) model of care and eligibility; (2) privacy and data protection; (3) program structure, outcomes, and evaluation; (4) educational content and teaching strategies; (5) timing, intensity, follow-up, and caregiver involvement; and (6) dignity, relational quality, and professional and organizational requirements. Recommendations supported a hybrid-by-default model with eligibility criteria, privacy-by-design using secure platforms and traceable documentation, structured programs with tailored multimodal content, staged pathways lasting 2–6 months after an initial in-person foundation, dignity-preserving options during remote encounters, professional training in communication and digital empathy, and integration into clinical planning and records. **Conclusions:** This consensus provides the first ostomy-specific, implementation-focused recommendations for standardizing remote patient education in Italy, with an emphasis on equity, privacy, dignity, evaluation, and workforce competencies.

## 1. Introduction

Living with an ostomy requires a sustained process of adaptation that extends beyond the physiological consequences of surgery. Individuals commonly face challenges related to body image, emotional well-being, intimacy, and social participation, which may reduce self-efficacy and compromise health-related quality of life [1,2,3,4,5]. In this context, structured patient education represents a core component of high-quality ostomy care, supporting skill acquisition, coping, and safe self-management. Educational pathways are ideally initiated in the preoperative period to promote realistic expectations and early learning and are continued after discharge to consolidate autonomy and long-term adaptation [6,7]. Consistent with this, the World Health Organization has emphasized the importance of person-centered, accessible, and continuous education for long-term conditions, enabling individuals and families to develop competencies for effective self-management [8,9].

In routine clinical practice, ostomy education is primarily delivered by specialist nurses (stomatherapists), whose advanced clinical and educational competencies are associated with improved outcomes, including higher self-efficacy, greater independence in stoma care, and better quality of life [10,11]. Traditionally, education has been provided in person using multimodal strategies such as demonstration, supervised practice, written materials, and follow-up, which have been shown to improve knowledge acquisition, adherence to care routines, and self-care behaviors, such as monitoring the condition and managing complications [11,12]. However, the expansion of digital health infrastructures, accelerated during the COVID-19 pandemic, has prompted increasing interest in remote educational models (e.g., videoconferencing and digital platforms) to preserve continuity of care and reduce logistical barriers.

Emerging evidence suggests that remote and telemedicine-based approaches to ostomy care may be effective, yet the literature remains heterogeneous in terms of intervention content, delivery channels, and outcome measurement. A recent scoping review described telemedicine interventions delivered via social and conferencing software, mobile applications, and remote devices, spanning clinical guidance and counseling, psychological and peer support, and remote follow-up; outcomes included quality of life, knowledge, self-efficacy, self-care ability, complications, satisfaction, and psychosocial indicators [13]. In a randomized controlled study, a post-discharge videoconferencing program delivered through structured sessions improved self-efficacy and adaptation to living with a stoma and increased the proportion of individuals performing independent stoma care [14]. In addition, a systematic review and meta-analysis reported a lower occurrence of stoma-related complications among adults receiving telemedicine interventions compared with usual care, suggesting a potential preventive effect when remote support is appropriately designed and implemented [15].

Despite these promising findings, implementation remains inconsistent, with limited guidance on how to structure, deliver, time, and evaluate remote educational pathways for people living with an ostomy. Key uncertainties include which components are essential (e.g., content and “dose” of education, follow-up intensity, optimal timing of administration), how to ensure equity and acceptability across varying levels of digital literacy and access, the professional competencies required, and how to document remote pathways within clinical records to ensure traceability and continuity. Remote educational pathways for people living with an ostomy remain under development and not yet standardized. While some stomatherapists have already incorporated remote education into routine care, others are planning to implement it in their clinical practice. In this early and evolving phase, and given the scarcity of ostomy-specific evidence, there is a clear need for shared, practice-oriented recommendations developed jointly by professionals (and informed by patient perspectives) to support the organization, delivery, and management of remote educational pathways. In areas where evidence is still developing, formal consensus methods can provide pragmatic direction for clinical practice and standardization [16]. The Delphi technique is particularly suitable for synthesizing expert judgment in contexts characterized by heterogeneous interventions and limited definitive evidence, enabling iterative refinement of statements and quantification of agreement across rounds [17].

In Italy, telemedicine has been systematically integrated into the National Health Service through national policy documents. The State–Regions Agreement of 17 December 2020 [18] established national guidelines for the provision of telemedicine services, while the Ministerial Decree of 21 September 2022 [19] approved additional guidelines defining functional requirements and service levels for telemedicine services. While these documents constitute a significant regulatory framework for incorporating remote care into routine healthcare delivery, they offer general guidelines and do not include disease- or condition-specific recommendations for the design, delivery, documentation, and evaluation of remote educational pathways for individuals living with an ostomy. This gap is particularly pertinent because ostomy care in Italy has traditionally depended on specialized nursing expertise and structured educational pathways. The joint position statement by the Italian Society of Surgery and the Association of Stoma Care Nurses on preoperative stoma siting emphasized that specialized preoperative counseling and appropriate stoma site marking should be guaranteed to all patients undergoing enterostomy or urostomy surgery, to prevent complications, enhance quality of life, and support improved health outcomes [20]. Similarly, the Italian Association of Stoma Care Healthcare Providers’ documents on ostomy care and best practices conceptualize ostomy management as an integrated care process involving patient education, prevention and early detection of complications, self-care support, follow-up, and continuity of care across settings [21,22]. However, despite general national telemedicine guidance and Italian recommendations for ostomy care, no specific national recommendations currently delineate how to implement remote education for individuals living with an ostomy in clinical practice. Therefore, a national modified Delphi consensus was deemed appropriate to develop shared, practice-oriented recommendations tailored to the Italian healthcare and stomatherapy context, while remaining aligned with international evidence and best-practice principles.

The objective of this national modified Delphi consensus is to synthesize expert judgment and evaluate the stability of responses across successive rounds to achieve consensus on the design, conduct, and implementation of a remote patient education pathway for people living with an ostomy in Italy. This study is intended for stomatherapists, ostomy services, and health organizations implementing remote educational pathways for people living with an ostomy.

## 2. Materials and Methods

### 2.1. Definition of the Delphi Method

A modified Delphi study combined with a consensus conference approach [23,24] was conducted to develop expert consensus on remote patient education for people living with an ostomy. The Delphi method is a structured consensus technique used to collect and refine expert judgments through sequential rounds, controlled feedback, and predefined consensus criteria [23,25,26,27]. This approach was considered appropriate because evidence on remote education in ostomy care remains limited and fragmented, requiring the integration of clinical expertise and experiential knowledge [23,25,26,27].

### 2.2. Description of the Process

The Delphi method was applied through two main phases. First, an exploratory qualitative phase included a targeted literature review and a synchronous stakeholder focus group to generate and refine preliminary statements. The second phase comprised two anonymous online surveys in which participants rated the relevance, clarity, and feasibility of the statements. Consensus was defined a priori as ≥75% agreement, calculated as the proportion of responses rated 3 or 4 on a four-point Likert scale.

### 2.3. Study Design

The study followed the Accurate Consensus Reporting Document (ACCORD) reporting guideline [28], and was conducted entirely in Italian because all participants were recruited in Italy.

To include the entire spectrum of opinion, a heterogeneous sample was used [29]. The panel included 11 members: six stomatherapists and five people living with an ostomy. The inclusion of both professional and experiential expertise was intended to support the development of person-centered and practice-oriented recommendations.

### 2.4. Participants and Eligibility Criteria

#### 2.4.1. Expert Panel Description

The panel comprised experts, defined by Goodman (1987) [25] and subsequently by McKenna (1994), as a group of informed individuals [26] and as ‘specialists’ in their respective fields [25], or individuals possessing knowledge about a specific subject. Consequently, the expert panel comprised stomatherapists, nurses with expertise in caring for people living with an ostomy and people living with an ostomy, who were considered experts by experience based on their lived experience of the condition. Stomatherapists were selected based on completion of a 1500 h post-bachelor specialization course in stoma care, the absence of conflicts of interest related to the research, and possessing at least 5 years of experience in the field. Individuals living with an ostomy were chosen in collaboration with the Italian Federation of Associations of Patients living with a Stoma or Incontinence (FAIS). They included both individuals with long-term experience living with an ostomy and newly diagnosed ostomates. People living with an ostomy were selected based on their high level of self-care, as assessed using the Ostomy Self-Care Index [30], their direct experience of the condition, and their voluntary willingness to participate in the Delphi study. The panel comprised six stomatherapists and five individuals living with an ostomy to reach a balance between heterogeneous expertise and feasibility [31]. The size of this Delphi study is consistent with Doughty’s recommendations [32]. All panelists participated voluntarily in the consensus process and did not receive any financial compensation. The final list of panelists was approved by the study’s chair and co-chair (GV, AP). All contacted panelists responded to the invitation and voluntarily agreed to participate in the study. Panelists were asked to declare potential conflicts prior to participation; none were identified.

#### 2.4.2. Recruitment Process

A snowball sampling method was employed to recruit the stomatherapists [33]. The recruiting process was initiated during an already scheduled online meeting with stomatherapists involved in research activities and related working practices in therapeutic patient education for people living with a stoma. Meanwhile, individuals living with an ostomy were recruited in collaboration with FAIS through an invitation letter sent via email.

#### 2.4.3. Research Steering Group

A research steering committee was constituted to undertake this study. Their responsibilities included preparing and disseminating materials for the Delphi rounds. This committee comprised two investigators and researchers with expertise in patient education, ostomies, and people living with an ostomy. The research steering committee did not participate in the Delphi rounds themselves; rather, they supervised and monitored the process. In particular, the researchers were the chair (GV) and the co-chair (AP) of the focus group and were, respectively, an assistant professor in nursing with a PhD and a research fellow in nursing and PhD student. Both researchers are nurses and conducted the entire Delphi study, from the focus group to the final round. The participants are reported in Table 1.

### 2.5. Procedures

**Figure 1:**
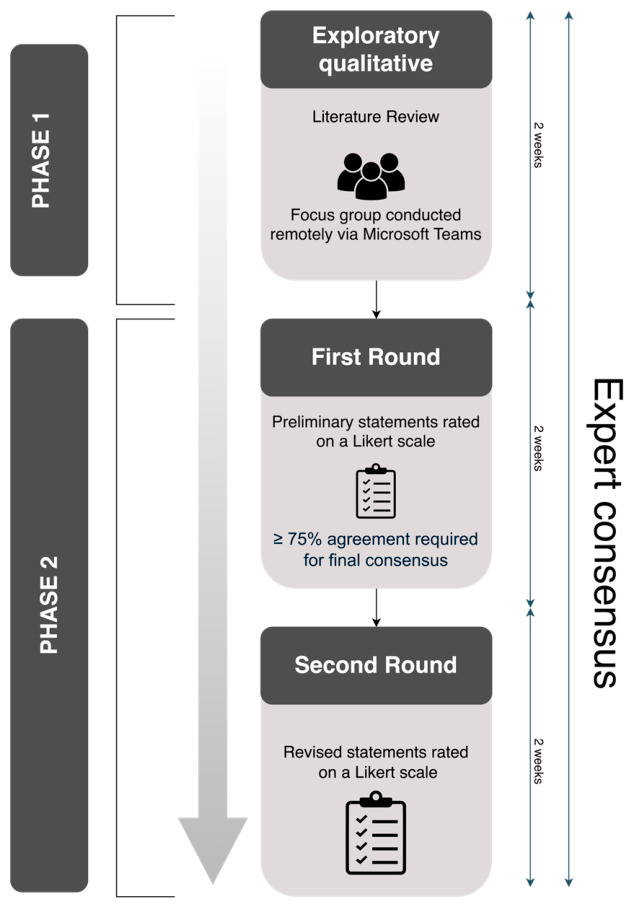
Flow diagram of the Delphi rounds.

The Delphi process was structured into two main phases. The first phase was exploratory and included the targeted literature review, preparation of the summary evidence document, and focus group discussion, which informed the development and refinement of the preliminary statements. The second phase comprised two anonymous online consensus rounds, in which panelists rated the statements using a four-point Likert scale. A pilot test was performed before administering the survey to ensure clarity and feasibility. The overall process lasted six weeks, with approximately two weeks allocated to each round, as shown in Figure 1.

#### 2.5.1. Phase 1: Exploratory Qualitative Phase

The initial exploratory phase was designed to generate, discuss, and refine the preliminary statements to be included in the subsequent Delphi consensus rounds. This phase included a targeted literature review, a synchronous stakeholder focus group, thematic refinement of the discussion, and the generation of the final set of statements.

First, a targeted literature review was conducted to identify available evidence on remote patient education and to support the development of the preliminary statements. The review focused on educational interventions, strategies, and recommendations delivered remotely or through digital, telehealth, or distance-based modalities. Given the limited availability of evidence specifically addressing remote education for people living with an ostomy, the steering committee also considered evidence from other chronic diseases or clinical conditions that offered relevant and transferable insights for remote patient education. The findings from this review were narratively synthesized and used to prepare a summary evidence document for the expert panel. This document included the preliminary statements, key definitions, the full reference list, and the level of evidence assigned to each statement according to the Johns Hopkins Nursing Evidence-Based Practice Model [34].

The exploratory phase then proceeded with a synchronous focus group, which was used as a brainstorming and item-generation session [29].

Panel members were asked to participate in a focus group [35], conducted remotely via Microsoft Teams [36]. This approach was employed to encompass a wide range of experiences and perspectives regarding the remote educational pathway.

Before the focus group, all participants received the summary evidence document to support reflection and discussion. Two researchers conducted the focus group: one served as the expert moderator (GV) and the other as the observer (AP). The expert moderator facilitated the discussion and ensured the group stayed on track with the agenda. Meanwhile, the observer’s responsibility was to closely monitor the session. The focus group began by registering participants to ensure that all were accounted for and had provided their consent. Once everyone was registered, the moderator introduced the focus group, outlined its purpose, and set ground rules for respectful communication. The discussion then proceeded with a series of carefully crafted statements sourced from the literature about remote patient education. Throughout the session, the moderator facilitated the conversation, ensuring all viewpoints were heard. After the discussion, the moderator summarized the key points raised and invited participants to share final reflections before they left the call. The research team then performed a thematic refinement of the feedback collected during the focus group. Comments and suggestions were reviewed, overlapping content was merged, unclear wording was revised, and new statements proposed by participants were incorporated when relevant. This process led to the generation of the set of statements that were included in the subsequent Delphi consensus rounds. In addition, panel members retained the opportunity to suggest new statements during the subsequent rating rounds.

##### Literature Review for the Summary Evidence Document

The preliminary literature review was conducted in accordance with the JBI Manual for Evidence Synthesis [37] and completed on 30 September 2025. The search was carried out in four electronic databases: MEDLINE via PubMed, CINAHL, Scopus, and Web of Science [Supplementary File: Literature review Search Strategies and PRISMA Flow Diagram]. The review aimed to identify and synthesize available evidence on remote patient education for people living with a stoma and to inform the development of preliminary consensus statements.

The search strategies combined, where available, indexed terms with free-text keywords related to three main concepts: enteral and urinary ostomies, patient education, and remote or distance-based care. Search terms included variations of “ostomy,” “stoma,” “colostomy,” “ileostomy,” “urostomy,” “patient education,” “health education,” “remote education,” “telehealth,” “distance education,” and “digital health.” Boolean operators and database-specific syntax were adapted for each database.

Studies were considered eligible if they addressed educational interventions, strategies, or recommendations delivered remotely or through digital, telehealth, or distance-based modalities and if their content was relevant to patient education. No timeframe filter was applied, ensuring that all pertinent records published about the topic could be found. Given the limited availability of studies specifically focused on people living with a stoma, evidence from other chronic diseases or clinical conditions was also considered when it provided transferable insights into remote patient education. Records that did not address patient education, did not involve remote or distance-based modalities, or were not available in full text were excluded.

Following database searching, records were deduplicated and screened using the Rayyan web application [38] for relevance by title and abstract, and potentially eligible papers were assessed in full text. A total of 32 pertinent papers were included. The evidence was synthesized narratively, with particular attention to recurring educational components, delivery modalities, the advantages and limitations of remote education, patient–professional communication, digital access, privacy, and the personalization of care. The findings were then used to draft and refine the preliminary statements submitted to the expert panel during the consensus process.

Overall, the review highlighted that only a limited number of available studies specifically addressed remote education in the ostomy field. Most of the included evidence referred to other chronic diseases or clinical conditions in which remote educational interventions had been implemented. This finding confirmed an important gap in the ostomy literature and supported the need for a consensus-based approach. In particular, the scarcity of condition-specific evidence suggests that recommendations for remote education in ostomy care cannot be directly transferred from other clinical fields without considering the unique needs of people living with a stoma, including appliance management, prevention and recognition of complications, body image concerns, privacy, stigma, and the need for individualized support from stomatherapists.

#### 2.5.2. Phase 2: Modified Delphi Consensus

##### Round 1

This round aimed to refine and prioritize the statements to structure the recommendations that emerged in the first round. The objective of the first round was to gather expert consensus on the relevance, feasibility, and clarity of the preliminary statements derived from the focus group data. These statements reflected the participants’ views on content, format, delivery methods, and evaluation strategies for remote educational interventions tailored to individuals living with an ostomy. In this way, this approach enabled the efficient and rapid collection of expert opinions while maintaining control over feedback [39]. Stomatherapists and expert participants from the first phase were invited to rate each statement using a four-point Likert scale (1 = strongly disagree; 4 = strongly agree). They were also given space to comment on each item and suggest rewording or additional elements as needed. This round was designed to take less than 30 min to complete [40].

The second round was conducted to finalize expert agreement on the key components of remote education for individuals living with an ostomy. The results and participant feedback from the previous round informed the design of this round.

This round aimed to reach definitive consensus on the revised statements that did not meet the agreement threshold in the first Delphi round and to validate the complete set of recommendations emerging from the overall process. This step marked the conclusion of the consensus-building phase, ensuring that the final framework reflected a shared vision among experts [23].

Participants received a summary of the first-round results, including aggregated ratings and a synthesis of qualitative comments. A new, streamlined survey was distributed by email, presenting the final versions of the revised statements that had previously lacked consensus, along with a comprehensive list of all statements that had reached consensus for final validation. The panelists received the full set of statements, including those that had already reached consensus in round one, and were allowed to re-rate all statements, again on a four-point Likert scale (1 = strongly disagree; 4 = strongly agree). Participants could also provide brief comments when clarification or endorsement was needed. Statements that reached ≥75% agreement were considered to have achieved final consensus. Participants were explicitly informed that this was the final round, and their feedback would shape the final set of recommendations for designing and implementing remote education for patients with ostomies. Quantitative data were analyzed to determine whether the revised items reached the predefined consensus threshold. Qualitative comments were reviewed to identify any remaining areas of ambiguity or concern, although no further modification was planned beyond this round.

The rounds of this Delphi study were conducted by administering two online surveys in Microsoft Forms and distributing them via email.

### 2.6. Data Analysis

The audio recording of the focus group was transcribed verbatim to capture any modifications to the statements proposed by the participants [41]. Similar or identical statements were combined, and all comments were organized to modify the statements based on the experts’ opinions. For each Delphi round, responses to each statement were analyzed descriptively using weighted frequencies and percentages across the four-point Likert scale, where 1 indicated the lowest level of agreement and 4 the highest. Agreement was defined as the proportion of weighted responses rated 3 or 4, and consensus was assessed against the predefined agreement threshold. For each statement, measures of central tendency and dispersion were calculated, including the weighted mean, median, and interquartile range. Changes between the two rounds were evaluated by comparing agreement percentages, mean scores, and dispersion indicators across rounds. Stability was interpreted as the absence of meaningful deterioration in agreement or dispersion between consecutive rounds, while improvements were identified when Round 2 showed higher agreement, higher mean scores, or reduced variability compared with Round 1.

### 2.7. Defined Agreement

The consensus threshold among the researchers was set at 75% (agree/strongly agree), as also outlined by Keeney et al. [42].

### 2.8. Anonymity

The expert panel guarantees confidentiality, noting that while respondents might recognize one another, their evaluations and opinions remain completely anonymous [26]. Complete anonymity was guaranteed in the second and third rounds, whereas the participants’ presence during the focus group did not allow for anonymity.

### 2.9. Rigor

To ensure methodological rigor, a minimum response rate of 70% was prespecified for each Delphi round. Response rates were 100% in both the first and second rounds.

### 2.10. Reliability

Several strategies were implemented to minimize bias and promote consistency, thereby ensuring the reliability of this modified Delphi study. Using a focus group in the first round helped generate relevant opinions from both professional and lived experiences. Retaining the same group of participants throughout all three rounds allowed for stable comparisons across responses [43]. Anonymity was maintained in the second and third rounds to avoid group pressure and influence [26]. The questionnaires were pretested to ensure clarity, and feedback from each round was shared with participants to guide the next round [31]. Although some researchers have questioned the reliability of Delphi studies due to potential personal and situational bias [44], this study’s clear, structured method with consistent panel selection, questionnaire design, and predefined agreement thresholds helped strengthen the reliability and quality of the results.

### 2.11. Validity

To strengthen the methodological rigor of the study, several strategies were adopted. The involvement of both expert stomatherapists and people living with an ostomy allowed the consensus process to integrate clinical and experiential perspectives on remote education. The exploratory phase, conducted through a focus group and informed by a targeted literature review, supported the generation and refinement of preliminary statements grounded in both available evidence and stakeholder experience. The subsequent Delphi rounds enabled anonymous rating, controlled feedback, and progressive refinement of the statements, supporting the development of shared consensus among participants. However, given the pragmatic and consensus-based nature of the study, the findings should not be interpreted as fully comprehensive or universally generalizable. Rather, they reflect the informed perspectives of a purposively selected panel within the Italian context. The inclusion of participants with different professional and experiential backgrounds may enhance the relevance and transferability of the findings, although transferability remains dependent on contextual similarity. To improve transparency and confirmability, the main steps of the study design, data collection, and analysis were documented and reported in line with recommendations on trustworthiness in qualitative research [45].

### 2.12. Ethics Statement

This study was conducted in accordance with the Declaration of Helsinki. Formal ethics committee approval was not sought because the study did not involve clinical interventions, pharmacological treatment, medical devices, biological samples, or the collection of clinical data from medical records. Participants were involved voluntarily only in relation to their professional expertise as stomatherapists or their lived experience as persons living with an ostomy, and no patient-level clinical or health-related data were collected. All participants received written information about the study and provided written informed consent before participation. Participation was entirely voluntary, confidentiality and anonymity were safeguarded, and participants could withdraw at any time without consequence. Data were collected and processed in compliance with Regulation (EU) 2016/679 and the Italian Privacy Code (Legislative Decree No. 196/2003), ensuring confidentiality, anonymity, and data minimization. The study was developed in partnership with FAIS, which supported stakeholder involvement and reviewed participant-facing materials for clarity and appropriateness. Clinical practice change and evidence base:

The final recommendations that reached consensus are reported in the following format:

Consensus statement:

- Level of evidence and quality using the Johns Hopkins Nursing Evidence-Based Practice Model [34].
- Risk of bias scoring: Assessed using different measurement tools based on the study’s design. In particular, for quantitative studies, we used the Mixed Methods Appraisal Tool (MMAT) [46]; for qualitative studies, we used the Critical Appraisal Skills Programme (CASP) checklist [47]; and we used the Risk Of Bias In Systematic Reviews (ROBIS) for reviews [48] [Supplementary File].
- Rationale and references from the literature.
- Clinical practice recommendations accompany each statement for potential adoption based on the aggregate strength of evidence:
  - Consider in your practice settings: This practice change should be considered based on a high level of evidence.
  - Expert opinion: The expert panel’s recommendations made for consideration based on clinical and real-world experience and common sense.

## 3. Results

During three Delphi rounds held between October and November 2025, the expert panel reached consensus on eight definitions of remote education (Table 2) and 14 statements regarding the design, delivery, and governance of remote educational pathways for individuals living with a stoma. To facilitate the use and the comprehension, the final statements were grouped into six domains: (1) model of care and eligibility; (2) privacy and data protection; (3) program structure and evaluation; (4) educational content and teaching strategies; (5) timing, intensity, follow-up and caregiver involvement; and (6) dignity, relational quality, and professional/organizational requirements. Collectively, these statements support a hybrid educational model combining remote and in-person sessions, with clear eligibility criteria to help reduce inequalities. They call for well-structured, measurable programs delivered through modular, multimodal educational strategies tailored to individual needs and levels of digital literacy. They also highlight the importance of appropriate timing, ongoing follow-up, and caregiver involvement when needed, alongside robust safeguards to protect privacy and dignity and to ensure professionals are adequately prepared, particularly in communication, digital empathy, and organizational documentation and traceability.

Table 3 summarizes the quantitative results of the Delphi rounds. For each statement, the table reports the weighted distributions of responses across the four-point Likert scale for Rounds 1 and 2, together with the corresponding agreement percentages and final consensus rates. Round 1 served as the baseline for comparison, while Round 2 was analyzed to assess the evolution and stability of consensus following statement refinement based on panelists’ feedback. Descriptive statistics, including the median and interquartile range, are also reported to describe the central tendency and dispersion of responses.

### 3.1. Statements

Table 4 describes the legend for the risk of bias interpretation of the statements.

#### 3.1.1. Domain 1: Model of Care and Eligibility

The panel agreed that remote education should be framed within an integrated model of care, prioritizing hybrid pathways (remote and in-person sessions) and applying eligibility criteria to avoid widening disparities. Education, regardless of modality, should be individualized to consider patients’ capabilities, baseline knowledge, and health literacy levels.

**Statement** **1.** *The use of educational pathways that include both remote and in-person modalities appears to be more effective for quality of life and adherence than modalities delivered only in person or only remotely.*

*Not all people with a stoma benefit equally from remote educational sessions: eligibility criteria (ability to make video calls, possession of electronic devices and an internet connection, etc.) and hybrid modalities (in-person and remote interventions) could reduce inequalities.*

[Level of evidence: III A; Risk of bias: ROBIS 🟠 some concerns [51]; Recommendation: Consider in your practice settings]

Hybrid educational pathways that integrate both in-person and remote components may present a practical advantage over single-modality education. This approach retains the benefits of face-to-face instruction, such as hands-on skills training, direct assessment, and the development of therapeutic relationships, while utilizing remote interactions for timely reinforcement, troubleshooting, and follow-up between interventions. From this perspective, enhancements in quality of life and adherence are behaviorally plausible, as repeated touchpoints can bolster self-efficacy, address emerging barriers promptly, and support sustained routines [51]. However, not all individuals with a stoma uniformly experience the benefits of remote sessions. Factors such as digital access and literacy, device and connectivity availability, and the capacity to engage in video calls may serve as ‘gatekeepers,’ potentially exacerbating disparities if not explicitly addressed. Consequently, establishing eligibility criteria and offering a hybrid option, rather than a remote-only approach, may mitigate inequalities by ensuring that patients who cannot reliably access telehealth still receive adequate education and monitoring.

**Statement** **2.** *In designing an educational pathway, whether in person or remote, one must always consider the patient’s type and abilities, their level of knowledge (health literacy), and any related specific needs.*

[Level of evidence: expert opinion]

The panel of experts convened to discuss this statement and concurred that educational pathways should be tailored to the individual, irrespective of whether they are delivered in person or remotely. Critical elements to evaluate in advance include the type of stoma and the patient’s functional abilities, as these factors directly influence the skills that can be acquired and how training should be administered. Additionally, it is imperative to consider the patient’s baseline knowledge and health literacy, as understanding health information affects engagement, proper technique, and long-term self-care. Finally, identifying specific needs, whether clinical, practical, or psychosocial, enables the pathway to address pertinent issues and select the most appropriate educational format, materials, and level of support.

#### 3.1.2. Domain 2: Privacy, Data Protection, and Enabling Conditions

The perceived security and confidentiality of remote encounters were a central enabling condition for participation. The panel recommended platforms that ensure privacy and data protection, and integrate with clinical documentation. Where this is not feasible, the use of organizational devices (rather than personal devices) was proposed as a mitigation.

**Statement** **3.** *Platforms that guarantee privacy, data protection, and integration with clinical documentation are recommended. If this is not possible, the use of organizational devices and not personal devices is suggested.*

[Level of evidence: expert opinion]

Educational pathways that use remote components should rely on platforms that ensure privacy, data protection, and the secure handling of health information, ideally integrated with clinical documentation to support traceability and continuity of care. This reduces the risks associated with data breaches, inappropriate sharing of sensitive content (e.g., images of the stoma/peristomal skin), and fragmented records. When institutional platforms are unavailable, using organizational devices (rather than personal smartphones or laptops) is a pragmatic safeguard: it supports standardized security settings, controlled access, and clearer accountability for the storage and transmission of patient data.

**Statement** **4.** *Implementing a remote educational pathway requires particular attention to protecting participants’ privacy.*

*The perception of security and confidentiality constitutes an essential prerequisite for adhering to this mode of education. Patients tend to participate only if they perceive that their personal data, images, and sensitive information shared during the session are managed securely and with defined rules.*

[Level of evidence: III (A-B); Risk of bias: CASP: 🟢 low risk [52,53] 🟠 some concerns [54], MMAT: 🟠 some concerns [55]; Recommendation: Consider in your practice settings]

Remote educational pathways should be implemented with explicit, visible safeguards for privacy and confidentiality, as these are not only legal/ethical requirements but also determinants of engagement and sustained participation. Qualitative evidence shows that online environments can foster a sense of anonymity and comfort, and online privacy may shape how openly participants interact (e.g., willingness to ask questions) [52]. At the same time, concerns about data safety and anonymity are consistently described as potential downsides of eHealth and can act as barriers to uptake if not proactively addressed [55].

Operationally, this supports the use of secure platforms with clear rules for managing personal data and any shared materials, alongside transparent consent processes and confidentiality assurances, as reported in online education research ethics procedures [52].

#### 3.1.3. Domain 3: Program Structure, Outcomes, and Evaluation

The panel described remote education as most appropriate when delivered as a structured program with explicit objectives, contents, modality, timing, and measurable indicators, preferably using available instruments. A two-part structure was recommended, comprising an initial phase to achieve/assess autonomy, followed by continued follow-up to reinforce behaviors and skills.

**Statement** **5.** *Educational sessions must be structured around objectives, content, delivery modality, evaluation indicators (measured with instruments, if available), and timing.*

*Indicators are intended, for example, to include: number and type of complications, level of self-care, and quality of life.*

[Level of evidence: I A; Risk of bias: ROBIS: 🟢 low risk [56]; Recommendation: Consider in your practice settings]

Educational sessions should be planned and reported as structured interventions, with clear objectives, core contents, delivery modality (in-person, remote, or blended), frequency and duration, and predefined evaluation indicators. This structure is important because evidence syntheses show that educational interventions are often poorly described, with unclear learning outcomes and heterogeneous reporting of outcomes, reflecting limitations that reduce reproducibility and make it difficult to compare effectiveness across studies [56]. Using measurable indicators, preferably assessed with validated instruments, strengthens evaluation and supports implementation decisions. In the included trials of patient education, outcomes commonly targeted domains such as quality of life, medication adherence and patient knowledge/skills, while clinical endpoints (e.g., disease activity, flare-ups) and healthcare use were measured less consistently [56].

**Statement** **6.** *It is important to structure a remote educational pathway, characterized by a first part aimed at assessing and developing autonomy, and a second part in which the person with a stoma continues to be cared for through follow-up.*

*Educational follow-ups are useful for reinforcing competencies in behaviors and skills.*

[Level of evidence: I B; Risk of bias: MMAT: 🟢 low risk [57]; Recommendation: Consider in your practice settings]

A two-step educational pathway comprising an initial phase aimed at fostering autonomy and skill acquisition, followed by a structured follow-up to sustain and reinforce competencies, is supported by the findings of Si et al. [57]. In their cluster-randomized trial, participants engaged in a structured, limited-duration (7 consecutive days) online program that incorporated daily quizzes to evaluate and consolidate learning. The outcomes were reassessed post-intervention, immediately and subsequently at 1 and 3 months [57]. Notably, the authors observed that motivation, behavioral skills, and willingness showed the most significant improvements after the intervention, although these gains tended to diminish over time. Si et al. explicitly highlighted the need for re-education to sustain educational impact, serving as a reinforcement phase to prevent skill attrition and consolidate behaviors following the initial autonomy-building phase [57].

#### 3.1.4. Domain 4: Educational Content and Teaching Strategies

Consensus highlighted the need for modular content covering core self-management domains, implemented according to individual needs, and delivered through multimodal resources (e.g., written materials, videos, images) tailored to digital literacy, particularly for older adults.

**Statement** **7.** *It is necessary to divide remote educational pathways into thematic modules, each dedicated to an area of management of the condition and implemented based on the individual’s needs, such as management of the stoma and peristomal skin, management of activities of daily living, nutrition, sexuality, recognition and management of complications, psychosocial support, and management of devices and supplies.*

*This modularity allows both gradual learning progression and the possibility of adapting contents to the ability level and preferences of the person with a stoma.*

[Level of evidence: III A; Risk of bias: ROBIS 🟠 some concerns [51]; Recommendation: Consider in your practice setting]

Remote educational pathways should be structured in a modular format, with each module concentrating on a distinct domain. This approach facilitates both progressive learning and personalization. Martínez-Miranda et al. examined patient education pathways that were delivered through various modalities (online, telephone, in-person, mixed), finding that their content was largely homogeneous, typically encompassing general knowledge about the disease, management knowledge, and essential self-management skills [51]. This finding suggests that education is most effective when organized around clear content areas and practical competencies, rather than being presented as unstructured information. A modular design enhances the delivery and adaptability of this content: modules can be selected and prioritized based on the individual’s needs, abilities, and preferences, and revisited over time if necessary [51].

**Statement** **8.** *Distance education pathways are more effective when delivered through a range of strategies and tools (written materials, videos, images), chosen and adapted based on the patient’s actual knowledge, access, and abilities.*

*Older age and low digital literacy necessitate personalizing the pathway.*

[Level of evidence: III C; Risk of bias: not applicable [58]; Recommendation: Consider in your practice setting]

Distance education pathways are more effective when they incorporate a variety of complementary tools, such as written text, images, videos, and quizzes, tailored to the individual’s health literacy and capabilities. In Hamid et al.’s study, the course was deliberately designed as a multimodal resource, recognizing that certain concepts are challenging to grasp through text alone [58]. Consequently, the authors employed diagrams, flow charts, illustrations, images, videos, written text, analogies, and quizzes to enhance comprehension and engagement. The same study emphasizes the necessity of personalization, noting that many adults possess limited reading proficiency and that patient materials are frequently composed at an excessively advanced level. To address this, the authors intentionally crafted content with simpler readability and engaged non-medical individuals and patients to review it for improved clarity. Furthermore, Hamid et al. highlight that advanced age and low digital literacy can impede the utilization of online education [58]. Therefore, remote educational pathways should incorporate practical adjustments and flexible formats to ensure accessibility, such as offering short versus long versions, accessibility features, and language support.

#### 3.1.5. Domain 5: Timing, Intensity, Follow-Up, and Caregiver Involvement

The panel agreed on the importance of ensuring an adequate duration and a progressive schedule for programs, recommending 2–6 months, with more frequent sessions initially, followed by subsequent follow-up sessions. When autonomy is not achieved, the inclusion of caregivers should be considered, provided it is feasible and acceptable. Remote delivery should commence following an initial in-person foundation, typically one month post-surgery, at the clinician’s discretion and with consideration of patient engagement.

**Statement** **9.** *The most effective remote educational pathways are characterized by a duration of 2 to 6 months, with more closely spaced initial sessions and subsequent follow-up sessions. If the person with a stoma is unable to achieve sufficient autonomy, consider including a caregiver in the pathway. If the caregiver is available and the person with a stoma allows it, the caregiver can take part in the educational pathway, regardless of the difficulty in achieving levels of autonomy.*

*This time frame appears sufficient to promote the progressive learning of stoma management skills (autonomous management of the stoma and peristomal skin, nutrition, etc.), consolidate problem-solving ability, and prevent the onset of complications (recognizing a complication, managing it, and contacting the stomatherapist). A shorter pathway risks not ensuring the stability of competencies.*

[Level of evidence: III A; Risk of bias: ROBIS 🟠 some concerns [51]; Recommendation: Consider in your practice setting]

The proposed intervention duration of 2–6 months, with more frequent sessions initially followed by subsequent follow-ups, aligns with the intervention patterns documented by Martínez-Miranda et al. [51]. Although the overall duration ranged from 1.5 to 18 months, most trials occurred over a multi-month timeframe, typically 3–6 months. Several programs integrated an initial educational phase with ongoing contacts in the subsequent months. This supports the notion that a brief, singular intervention may be inadequate for maintaining competency stability, whereas a multi-month structure facilitates progressive learning, consolidation, and reinforcement. The panel of experts approved reducing the duration range to 2–6 months, which is considered optimal, occurring shortly after the surgical procedure, when newly ostomized individuals are ready to begin building their knowledge and skills.

**Statement** **10.** *The remote educational pathway can start after in-person educational interventions, at least 1 month after the surgical intervention and in any case at the professional’s discretion, considering the engagement level of the newly ostomized person. This facilitates getting to know the educational figures involved in the pathway, building trust, and presenting the educational pathway.*

[Level of evidence: III A; Risk of bias: MMAT: 🟢 low risk [59]; Recommendation: Consider in your practice setting]

A remote educational pathway may be more acceptable and effective when it starts after an initial in-person phase, once the person has had time to recover and build a basic relationship with the care team. Evidence from Gusdorf et al. supports the value of preparatory contact before telehealth: a structured telephone call conducted 1–5 days prior to the video sessions significantly increased the likelihood of a successful audio-visual telehealth encounter (OR 0.54; 95% CI 0.48–0.60) [59].

Early “set-up” moments where the pathway is explained, expectations are clarified, and practical barriers are addressed are key enablers of engagement.

In Gusdorf et al., the preparatory calls helped patients understand what telehealth is, supported technical readiness (apps, portal access, test visit), and created an opportunity to answer questions and reduce confusion [59].

Translating this to stoma education, initiating remote sessions after an initial in-person period (e.g., after the first month post-surgery) can facilitate introducing the educational team and building trust, as well as ensuring the patient is “ready” for remote follow-up, both technically and motivationally. Importantly, Gusdorf et al. also highlight that certain groups (e.g., older adults) are at higher risk of unsuccessful video visits, reinforcing the need for professional discretion and readiness assessment when starting remote follow-up [59].

#### 3.1.6. Domain 6: Dignity, Relational Quality, Access, and Professional-Organizational Requirements

The panel recognized that remote modalities can improve access by reducing logistical barriers, but should also protect dignity, including managing embarrassment/shame related to body exposure and remote interaction through practical alternatives (e.g., patient-shared photographs, control of camera framing). In parallel, the panel highlighted the need for training not only in technology use but also in communication and digital empathy, and for embedding remote pathways within clinical planning and documentation to ensure traceability and continuity.

**Statement** **11.** *Consider the possibility that, although the person has given consent to the remote educational pathway and therefore to showing themselves on webcam, the person may experience embarrassment/shame related to the body and the condition, to the device, and to their remote exposure. The possibility of offering alternatives (e.g., photographs sent by the patient or control over the camera framing) appears fundamental to guarantee dignity and reduce the experience of embarrassment/shame.*

[Level of evidence: expert opinion]

Experts from the panel agreed that even when a patient formally consents to remote education and webcam use, clinicians should anticipate that embarrassment or shame may still emerge, especially when the session involves body exposure, the stoma device, or discussing intimate aspects of daily management. This is clinically relevant because shame can reduce openness, limit participation, and ultimately undermine learning and adherence.

For this reason, the pathway should explicitly protect dignity by offering practical alternatives that keep the patient in control. Examples include allowing the patient to avoid showing the stoma live and instead share photographs if they prefer, agreeing in advance on camera framing (e.g., face-only unless the patient decides otherwise), using stepwise exposure (first talk and education, then optional visual assessment), and ensuring the patient can pause/stop video at any time without consequence. These options make consent “usable” in real life, reduce perceived vulnerability, and support participation while respecting personal boundaries.

**Statement** **12.** *The delivery of distance education programs is associated with a reduction in logistical barriers, for example, related to travel, time, and the limited availability of places in in-person pathways.*

[Level of evidence: III B; Risk of bias: ROBIS: 🔴 high risk [60]; Recommendation: Consider in your practice setting]

Distance educational programs can reduce logistical barriers because they limit (or eliminate) the need for travel, reduce time costs for patients and caregivers, and ease capacity constraints linked to on-site services.

Although Guirado-Fuentes et al. did not focus on education per se, they summarize evidence that telehealth-related models are promoted and evaluated partly because they avoid healthcare-related travel events (e.g., road/air travel), with implications for both access and resource use [60]. In this literature, reducing travel is treated as a concrete, measurable advantage of remote care delivery (including avoided journeys and associated burdens) [60].

**Statement** **13.** *Professionals should receive not only technical training (e.g., the use of technological devices), but also communicative and relational training to effectively manage the dual clinical and digital dimensions of the sessions. Professionals should develop “digital empathy” competencies for conducting remote educational pathways by actively using tools (screen sharing, emotion recognition, maintaining gaze at the camera, etc.). People with a stoma perceive this competence as decisive for the quality of the remote educational relationship.*

[Level of evidence: III B; Risk of bias: MMAT: 🟢 low risk [61]; Recommendation: Consider in your practice setting]

Professionals who deliver remote educational programs require competencies that extend beyond mere platform use. Kritsotakis et al. demonstrate that nurses’ eHealth literacy encompasses the ability to locate, comprehend, utilize, and critically assess online information to make informed health decisions [61]. The authors found that nurses reported the lowest confidence in using internet information for health decision-making, indicating that training should not be confined to technical skills but should also enhance judgment, communication, and relational practices in digital environments, enabling clinicians to translate digital information into safe guidance and sustain high-quality educational relationships. The same study also notes that both professionals and lay users may struggle to distinguish reliable from unreliable online information, which can directly affect the quality of care. In practice, this supports structured training that integrates (1) digital competence and information appraisal, and (2) communication skills tailored to remote interactions, such as clear explanations, checking understanding, and responding to emotions. These are the foundations that enable “digital empathy” behaviors, such as attentive presence and recognition of cues, to be effective and credible in remote education [61].

**Statement** **14.** *Remote educational pathways should be recognized and included in clinical planning and documentation to ensure traceability, continuity of care, and formal recognition of the pathway.*

[Level of evidence: III B; Risk of bias: MMAT: 🟠 some concerns [62]; Recommendation: Consider in your practice setting]

Remote educational pathways should be formally integrated into clinical planning and documented in the health record to ensure traceability, continuity, and organizational legitimacy.

A core component of Coffey et al.’s remote patient monitoring model is that patient-generated data are transmitted to a hub integrated within the electronic health record (EHR), which nurses use to provide individualized assessment, education, and care planning [62].

Importantly, the authors state that documentation occurs within the EHR, and that the program relies on standardized protocols and documentation standards to ensure regulatory compliance, quality reporting, and scalable implementation [62].

## 4. Discussion

This consensus conference offers a structured and implementation-focused framework for developing remote and hybrid educational pathways for individuals living with an ostomy. Across the different domains, the statements converge on the unified message that remote education should not be regarded as a standalone replacement for in-person care, but rather as one component of a broader, planned, and person-centered approach to stomatherapy education. This is particularly important in ostomy care, where education involves not only the transmission of information but also the acquisition of practical self-care skills, the management of intimate body-related issues, and the progressive adaptation to a life-changing condition. Therefore, the framework does not emphasize whether remote education should be used, but rather how it can be delivered responsibly, safely, and effectively within routine stomatherapy services.

To our knowledge, this is the first expert consensus specifically addressing remote education for individuals living with an ostomy. This represents an important contribution because specific evidence on ostomy care remains limited, despite the increasing investigation of digital health and remote patient education in chronic conditions [63]. Previous literature has highlighted the potential of eHealth platforms to support ostomy self-care, self-monitoring, interaction with stomatherapy nurses, and timely access to differentiated care; however, available studies have mainly focused on identifying desirable contents or technological functionalities rather than defining an integrated educational pathway for clinical implementation [13,14]. In line with this, ostomy-specific literature has also emphasized the potential contribution of digital applications and nursing guidance for the prevention and treatment of intestinal peristomal skin complications [64,65]. This consensus addresses this gap by translating broader evidence on remote education into practical recommendations tailored to the specific needs of people living with an ostomy.

This study’s innovative aspect lies not only in its ostomy-specific focus but also in the integration of professional expertise and patient experience. The involvement of expert stomatherapists with extensive clinical experience was crucial for interpreting evidence derived from other clinical areas and adapting it to the relational, technical, and emotional complexity of ostomy care. At the same time, including individuals living with an ostomy enhanced the practical relevance of the recommendations. Their contributions helped refine and, in some cases, reconsider specific statements to ensure that remote education was not designed solely from an organizational or professional perspective but also from the perspectives of feasibility, acceptability, privacy, dignity, and everyday life.

A central contribution of the consensus is its preference for hybrid educational pathways that integrate in-person and remote sessions. This finding confirms evidence from related fields suggesting that mixed or blended modalities may be particularly appropriate when patient education requires both direct interaction and flexible follow-up. For example, studies on patient education in breast cancer survivorship have suggested that mixed educational modalities may be promising for supporting quality of life and self-management, although the evidence remains cautious [51]. Similarly, studies conducted in chronic disease contexts have shown that remote or virtual education may reduce travel burden and improve accessibility, but often requires adequate technological support and careful adaptation of group processes [53]. In ostomy care, this hybrid approach appears especially appropriate. In-person encounters remain essential for hands-on skills training, direct observation of appliance changes, peristomal skin assessment, and relationship building, whereas remote encounters can support timely reinforcement, troubleshooting, and follow-up between visits.

The consensus expands previous literature by specifying when and how remote education may be most appropriately used in the ostomy pathway. For example, remote sessions may be particularly useful after hospital discharge, when patients’ needs evolve regarding appliance management, leakage, skin irritation, dietary adjustments, supply issues, or emotional adaptation. In practical terms, a hybrid pathway could include an initial in-person session focused on stoma assessment and basic appliance management, followed by scheduled remote sessions to review self-care progression, reinforce problem-solving strategies, and identify early signs of complications. This approach is consistent with remote patient monitoring models in which nurses use digital tools to provide individualized assessment, education, clinical planning, and follow-up while maintaining continuity of care [62,66].

Equity emerged as a transversal issue across the consensus domains. The recommendations confirm previous findings showing that digital education can improve access but may also reproduce or widen inequalities when digital literacy, connectivity, device availability, language, sensory limitations, or privacy at home are not adequately considered. Evidence from telehealth implementation has shown that preparatory support, such as structured pre-visit calls, may improve the likelihood of successful video visits and reduce gaps in use among vulnerable groups [59]. In this sense, the consensus extends the literature by proposing eligibility criteria as safeguards to ensure that remote education is appropriate, accessible, and acceptable for each person. Practical solutions may include pre-session technical checks, caregiver involvement when appropriate, simplified written instructions, telephone alternatives, and the option to switch from remote to in-person care when clinical or personal needs require it.

Privacy, dignity, and data protection were also identified as enabling conditions for participation. This domain is particularly relevant in ostomy care because remote education may involve discussing or sharing sensitive information, images, or body exposure. The consensus confirms broader concerns about the acceptability of eHealth interventions, in which perceived usefulness, digital confidence, effort expectancy, and social influence can affect engagement [55]. However, it expands these findings by emphasizing the specific vulnerability associated with stoma exposure during remote encounters. Practical strategies may include patient-controlled camera framing, stepwise exposure, use of photographs shared voluntarily by the patient, explicit consent before visual assessment, and secure platforms integrated with clinical documentation. Where institutional platforms are unavailable, the use of organizational rather than personal devices may strengthen governance, accountability, and confidentiality.

Another important contribution concerns the need to implement remote education as a structured program rather than as a set of sporadic or informal interactions. This recommendation aligns with systematic review evidence showing that patient education interventions are often heterogeneous and insufficiently described, limiting reproducibility and the interpretation of effectiveness. For instance, Gordon et al.’s review on patient education in inflammatory bowel disease found substantial variability in educational formats and limited reporting of intervention components, concluding that future research should better describe educational content, delivery methods, and outcomes [56]. Our consensus responds to this methodological and practical gap by recommending clear objectives, defined content, delivery modalities, timing, documentation, follow-up, and measurable indicators, preferably using validated instruments.

From an organizational perspective, implementation may be limited by several barriers, including a lack of dedicated time, insufficient staffing, inadequate digital infrastructure, the absence of shared documentation systems, limited reimbursement mechanisms, and variability in professionals’ digital competence. These barriers suggest that remote ostomy education cannot depend solely on the motivation of individual stomatherapists. Health organizations should provide secure digital platforms, institutional devices, professional training, technical support for patients, and clear protocols defining responsibilities, escalation criteria, and documentation standards. In addition, nurses’ eHealth literacy should be considered a prerequisite for safe and effective implementation, as previous evidence has shown that digital competence among nurses is associated with the practice environment and should be integrated into continuing professional education initiatives.

The findings also have implications for health policy. Remote and hybrid ostomy education should be recognized as part of routine stomatherapy services rather than as an emergency or exceptional solution. This requires policy-level support for reimbursement, integration with EHRs, quality indicators, professional training, and equitable access to digital care. Moreover, it is necessary to consider the potential environmental and organizational benefits of remote care. Reducing unnecessary travel and optimizing follow-up pathways may contribute to more sustainable healthcare delivery, although environmental impact should be systematically evaluated rather than assumed. Current debates in health technology assessment increasingly recognize environmental sustainability as a relevant dimension of healthcare value, although methods for incorporating it remain underdeveloped [60].

Overall, this consensus confirms the potential of remote education to support continuity, accessibility, and self-management in chronic care, while diverging from overly technology-centered perspectives that present remote delivery as inherently beneficial. In the ostomy context, remote education is appropriate only when embedded within a hybrid, individualized, privacy-conscious, and professionally governed pathway. Therefore, the resulting framework provides a pragmatic basis for clinical services, researchers, and policymakers seeking to implement remote patient education in a way that is not only technologically feasible but also clinically meaningful, equitable, and respectful of the lived experience of people with an ostomy.

### 4.1. Limitations

When interpreting these consensus statements, it is important to consider their scope and intended use. As expected in Delphi methodologies, the findings reflect the collective judgment of a multidisciplinary panel and should therefore be understood as practice-informed guidance rather than direct evidence of effectiveness. While the inclusion of individuals living with an ostomy strengthened the relevance and applicability of the statements, further work is needed to explore their transferability across different ages, stoma types, cultural backgrounds, socioeconomic conditions, levels of digital literacy, and geographical contexts. Since the consensus was developed within the Italian healthcare and stomatherapy context, external validation in other settings would further support its broader applicability. Finally, although the process was structured and iterative, the initial focus group may have been influenced by typical group dynamics, and future studies should further refine shared outcome definitions, thresholds, and core measures. Overall, these limitations identify important directions for future research and provide a basis for strengthening, validating, and implementing the consensus framework in clinical practice.

### 4.2. Implications

#### 4.2.1. Clinical Implications

The implementation of remote or hybrid stoma education should translate the consensus statements into routine, standardized care pathways. A hybrid-by-default model, complemented by clearly defined alternatives for individuals who cannot access or do not wish to use video consultations, may help maximize accessibility while reducing the risk of digital exclusion. Remote education should be delivered through a modular curriculum supported by multimodal materials, including written resources, images, and videos, tailored to patients’ health literacy, digital literacy, and functional abilities. Educational pathways should follow a staged schedule, ideally beginning with an in-person encounter to support engagement and the therapeutic alliance, followed by remote autonomy-building, structured reinforcement, and follow-up over 2–6 months. To ensure continuity of care, the clinical record should document remote sessions, educational content delivered, patient progress, and relevant outcomes.

#### 4.2.2. Organizational Implications

Healthcare organizations implementing remote stoma education should establish clear governance procedures for digital platforms, informed consent, documentation, and the secure management of sensitive materials, including photographs. A privacy-by-design approach should guide the selection and use of remote communication tools, with preference for organizational devices and institutional platforms when needed to ensure security, accountability, and traceability. Implementation also requires integration with existing care pathways, workload planning, and alignment with local documentation systems, including electronic health records. Therefore, future studies should evaluate organizational feasibility, resource requirements, workload implications, and costs associated with remote and hybrid education models.

#### 4.2.3. Implications for Health Policy

The findings support the need for policy-level guidance to standardize remote and hybrid stoma education within broader telemedicine frameworks. Future policy efforts should define minimum requirements for reporting, delivering, and evaluating remote education, including intervention components, dosage, timing, delivery mode, provider roles, and fidelity criteria. The development of a core outcome set, including standardized complication taxonomies, patient-reported outcomes, and equity indicators, would improve comparability across services and future studies. Pragmatic evaluations comparing hybrid and single-modality approaches are also needed, particularly among groups at greater risk of digital vulnerability.

#### 4.2.4. Implications for Professional Training

Implementing remote stoma education requires targeted workforce development. Professionals should receive training not only in the technical use of digital tools, but also in remote communication, dignity-preserving practices, privacy management, and digital empathy. The expert panel emphasized the pivotal role of patient associations in defining the relational and communication components of such training. Their involvement may help ensure that educational programs integrate patients’ perspectives on digital tools, image sharing, camera use, and therapeutic communication. Future research should define and measure digital empathy competencies and evaluate whether related training improves patient-reported experience, adherence, self-care, and clinical outcomes.

## 5. Conclusions

This consensus is the first on remote education for individuals living with an ostomy, offering a pragmatic, person-centered framework for implementing remote and hybrid education in ostomy care. The consensus highlights the perceived importance of hybrid-by-default models, structured and measurable educational programs, modular and multimodal content, and safeguards for privacy and dignity as key considerations for routine practice. The integration of remote encounters into clinical planning and documentation may support traceability and continuity of care, while professional training in communication and digital empathy may help preserve relational quality in virtual settings. Overall, these statements provide a preliminary, scalable framework to support greater standardization of remote ostomy education and to guide future empirical research evaluating feasibility, effectiveness, equity, and patient-centered outcomes.

## Figures and Tables

**Table 1:** Panelists’ details (listed in alphabetical order).

Panel Member	Italian Region	Stomatherapist	Individual with a Stoma	Methodologist
Chair: GV	Lombardia			X
Co-chair: AP	Lombardia			X
S1	Campania	X		
S2	Lombardia	X		
S3	Lombardia	X		
S4	Piemonte	X		
S5	Sardegna	X		
S6	Lazio	X		
I7	Lombardia		X	
I8	Lombardia		X	
I9	Lombardia		X	
I10	Lombardia		X	
I11	Lombardia		X	

**Table 2:** Definitions.

Term	Definition	References
Telemedicine	Remote delivery of healthcare services. It uses information, communication, and educational technologies for the education of patients and caregivers and for consultation among professionals.	WHO, 2024 [49]
Therapeutic education	A set of essential educational activities for the management of chronic conditions, carried out by healthcare professionals in educational processes in the care setting, designed to help a patient (or a group of patients and their families) manage adaptation to a new condition and prevent avoidable complications, while maintaining or improving quality of life. What specifically characterizes it is that it produces an additional therapeutic effect compared with that deriving from all other interventions (pharmacological, physiotherapeutic, etc.)	WHO, 1998; WHO Regional Office for Europe, 2023 [8,9]
In-presence education	Education in which healthcare professionals and patients/caregivers interact within the same environment, fostering empathic communication, mutual observation, and experiential learning.	Experts’ opinion
Remote education	Education in which healthcare professionals and patients/caregivers are not present in the same environment. A technological device is used for the contact (dedicated platforms, devices such as smartphones and tablets).	COMMISSION STAFF WORKING DOCUMENT Accompanying the Document Communication from the Commission to the European Parliament, the Council, the European Economic and Social Committee and the Committee of the Regions Digital Education Action Plan 2021–2027 Resetting Education and Training for the Digital Age, 2020 [50]; Experts’ opinion
Mixed modality education	Education that is delivered by providing educational sessions both in person and remotely, combining direct, relational interaction with the flexibility of remote learning.	Experts’ opinion
Follow-up in the field of stomatherapy	It is the structured and continuous set of scheduled meetings (in person or remotely) between the person with a stoma, the caregiver, and the stomatherapist, aimed at ensuring continuity of care and assistance after the surgery. It includes: periodic assessment of the stoma, the mucocutaneous junction, and the peristomal skin; assessment and adaptation of the appliances; prevention/management of complications; reinforcement of self-care skills and education; psychosocial support and quality of life; documentation of outcomes and, if necessary, activation of other professionals/services.	Experts’ opinion
Remote educational pathway in the field of stomatherapy	A set of both synchronous and asynchronous educational sessions, conducted by a stomatherapist, aimed at promoting autonomy in ostomy management, preventing complications, and ensuring continuity of care.	Experts’ opinion
Educational intervention	An educational session (synchronous or asynchronous) that is part of the educational pathway, focused on a specific topic.	Experts’ opinion

**Table 3:** Quantitative results of Delphi rounds.

Round	Statement	N Valid Anwers	% Likert 1	% Likert 2	% Likert 3	% Likert 4	Agreement n (3 + 4)	Mean	Median	IQR	Δ Agreement vs. Round 1	Δ Mean vs. Round 1	Δ IQR vs. Round 1	Interpretation
Round 1	Background	11	0.0%	0.0%	63.6%	36.4%	11	3.36	4.50	0.50				Baseline
Round 2	Background	11	0.0%	0.0%	0.0%	100.0%	10	4.00	5.00	0.00	0.00	0.64	−0.50	Improved
Round 1	Telemedicine	11	0.0%	0.0%	18.2%	81.8%	11	3.82	4.50	0.50				Baseline
Round 2	Telemedicine	11	0.0%	0.0%	0.0%	100.0%	11	4.00	5.00	0.00	0.00	0.18	−0.50	Improved
Round 1	Therapeutic Education	11	0.0%	0.0%	27.3%	72.7%	11	3.73	4.50	0.50				Baseline
Round 2	Therapeutic Education	11	0.0%	0.0%	9.1%	90.9%	11	3.91	4.50	0.50	0.00	0.18	0.00	Improved
Round 1	In presence education	11	0.0%	0.0%	18.2%	81.8%	11	3.82	4.50	0.50				Baseline
Round 2	In presence education	11	0.0%	0.0%	0.0%	100.0%	11	4.00	5.00	0.00	0.00	0.18	−0.50	Improved
Round 1	Remote education	11	0.0%	0.0%	18.2%	81.8%	11	3.82	4.50	0.50				Baseline
Round 2	Remote education	11	0.0%	0.0%	0.0%	100.0%	11	4.00	5.00	0.00	0.00	0.18	−0.50	Improved
Round 1	Mixed modality education	11	0.0%	0.0%	18.2%	81.8%	11	3.82	4.50	0.50				Baseline
Round 2	Mixed modality education	11	0.0%	0.0%	0.0%	100.0%	11	4.00	5.00	0.00	0.00	0.18	−0.50	Improved
Round 1	Follow-up in the field of stoma therapy	11	0.0%	0.0%	18.2%	81.8%	11	3.82	4.50	0.50				Baseline
Round 2	Follow-up in the field of stoma therapy	11	0.0%	0.0%	9.1%	90.9%	11	3.91	4.50	0.50	0.00	0.09	0.00	Improved
Round 1	Remote educational pathway in the field of stoma therapy	11	0.0%	0.0%	27.3%	72.7%	11	3.73	4.50	0.50				Baseline
Round 2	Remote educational pathway in the field of stoma therapy	11	0.0%	0.0%	0.0%	100.0%	11	4.00	5.00	0.00	0.00	0.27	−0.50	Improved
Round 1	Educational intervention	11	0.0%	0.0%	18.2%	81.8%	11	3.82	4.50	0.50				Baseline
Round 2	Educational intervention	11	0.0%	0.0%	18.2%	81.8%	11	3.82	4.50	0.50	0.00	0.00	0.00	Stable
Round 1	Statement 1	11	0.0%	0.0%	9.1%	90.9%	11	3.91	4.50	0.50				Baseline
Round 2	Statement 1	11	0.0%	0.0%	0.0%	100.0%	11	4.00	5.00	0.00	0.00	0.09	−0.50	Improved
Round 1	Statement 2	11	0.0%	0.0%	0.0%	100.0%	11	4.00	5.00	0.00				Baseline
Round 2	Statement 2	11	0.0%	0.0%	0.0%	100.0%	11	4.00	5.00	0.00	0.00	0.00	0.00	Stable
Round 1	Statement 3	11	0.0%	0.0%	9.1%	90.9%	11	3.91	4.50	0.50				Baseline
Round 2	Statement 3	11	0.0%	0.0%	9.1%	90.9%	11	3.91	4.50	0.50	0.00	0.00	0.00	Stable
Round 1	Statement 4	11	0.0%	0.0%	0.0%	100.0%	11	4.00	5.00	0.00				Baseline
Round 2	Statement 4	11	0.0%	0.0%	0.0%	100.0%	11	4.00	5.00	0.00	0.00	0.00	0.00	Stable
Round 1	Statement 5	11	0.0%	0.0%	0.0%	100.0%	11	4.00	5.00	0.00				Baseline
Round 2	Statement 5	11	0.0%	0.0%	0.0%	100.0%	11	4.00	5.00	0.00	0.00	0.00	0.00	Stable
Round 1	Statement 6	11	0.0%	0.0%	18.2%	81.8%	11	3.82	4.50	0.50				Baseline
Round 2	Statement 6	11	0.0%	0.0%	0.0%	100.0%	11	4.00	5.00	0.00	0.00	0.18	−0.50	Improved
Round 1	Statement 7	11	0.0%	0.0%	27.3%	72.7%	11	3.73	4.50	0.50				Baseline
Round 2	Statement 7	11	0.0%	0.0%	0.0%	100.0%	11	4.00	5.00	0.00	0.00	0.27	−0.50	Improved
Round 1	Statement 8	11	0.0%	0.0%	0.0%	100.0%	11	4.00	5.00	0.00				Baseline
Round 2	Statement 8	11	0.0%	0.0%	0.0%	100.0%	11	4.00	5.00	0.00	0.00	0.00	0.00	Stable
Round 1	Statement 9	11	0.0%	0.0%	9.1%	90.9%	11	3.91	4.50	0.50				Baseline
Round 2	Statement 9	11	0.0%	0.0%	0.0%	100.0%	11	4.00	5.00	0.00	0.00	0.09	−0.50	Improved
Round 1	Statement 10	11	0.0%	0.0%	45.5%	54.5%	11	3.55	4.50	0.50				Baseline
Round 2	Statement 10	11	0.0%	0.0%	9.1%	90.9%	11	3.91	4.50	0.50	0.00	0.36	0.00	Improved
Round 1	Statement 11	11	0.0%	45.5%	0.0%	54.5%	6	3.09	4.00	1.00				Baseline
Round 2	Statement 11	11	0.0%	0.0%	0.0%	100.0%	11	4.00	5.00	0.00	45.45	0.91	−1.00	Improved
Round 1	Statement 12	11	0.0%	0.0%	0.0%	100.0%	11	4.00	5.00	0.00				Baseline
Round 2	Statement 12	11	0.0%	0.0%	0.0%	100.0%	11	4.00	5.00	0.00	0.00	0.00	0.00	Stable
Round 1	Statement 13	11	0.0%	0.0%	9.1%	90.9%	11	3.91	4.50	0.50				Baseline
Round 2	Statement 13	11	0.0%	0.0%	0.0%	100.0%	11	4.00	5.00	0.00	0.00	0.09	−0.50	Improved
Round 1	Statement 14	11	0.0%	0.0%	0.0%	100.0%	11	4.00	5.00	0.00				Baseline
Round 2	Statement 14	11	0.0%	0.0%	0.0%	100.0%	11	4.00	5.00	0.00	0.00	0.00	0.00	Stable

Legenda: Baseline: Statement as created for Round 1; Stable: Statement remained stable after round 1; Improved: Statement improved in its agreement after round 1.

**Table 4:** Legend for risk of bias interpretation.

**🟢**	Low risk
**🟠**	Some concerns
**🔴**	High risk of bias
**⚪**	Unclear
*N/A*	Not applicable

## Data Availability

The data presented in this study are available from the corresponding author upon reasonable request. The data are not publicly available due to privacy and ethical restrictions.

## Supplementary Materials

The following supporting information can be downloaded at: https://www.mdpi.com/article/10.3390/nursrep16060203/s1, Supplementary File: Literature review Search Strategies and PRISMA Flow Diagram (Table S1. Search Strategies; Figure S1. PRISMA Flow Diagram 2020); Risk of Bias Assessment (Table S2. Quantitative studies (MMAT); Table S3. Qualitative studies (CASP qualitative; Table S4. Reviews (ROBIS); Table S5. Paper not assessable).

## Public Involvement Statement

Representatives of FAIS—Federazione Associazioni Incontinenti e Stomizzati (Italian National Federation of Associations of Incontinence and Ostomy Patients)—contributed to the development of this study by reviewing the protocol and participant-facing materials prior to study initiation and remained involved throughout the study. Their input helped enhance the relevance, clarity, and acceptability of the study design and materials for the target population. FAIS was not involved in the entire process and contributed to the interpretation of the findings and the discussion. FAIS was also involved in the decision to submit the manuscript for publication.

## Guidelines and Standards Statement

This manuscript was drafted in accordance with the ACCORD (*ACcurate COnsensus Reporting Document*) guideline for reporting consensus methods in biomedicine [28]. This guideline was used because the study adopted a modified Delphi consensus approach.

## Abbreviations

The following abbreviations are used in this manuscript:

ACCORD	Accurate Consensus Reporting Document
CASP	Critical Appraisal Skills Programme
EHR	Electronic Health Record
EU	European Union
FAIS	Federazione Associazioni Incontinenti e Stomizzati
GDPR	General Data Protection Regulation
MMAT	Mixed Methods Appraisal Tool
ROBIS	Risk Of Bias In Systematic reviews
WHO	World Health Organization
