# Supplementary material for: Remote Patient Education for People Living with an Ostomy: An Italian Expert Consensus Using a Modified Delphi Method
Source: Nurs Rep. 2026 Jun 15;16(6):203. doi: 10.3390/nursrep16060203 (PMC13304563; doi:10.3390/nursrep16060203)
Supplement: Supplementary file 1 [file nursrep-16-00203-s001.zip › nursrep-4302196-supplementary.pdf]

Remote Patient Education for People Living with an Ostomy: An Italian Expert Consensus Using a Modified Delphi Method

Supplementary file

Literature review Search Strategies and PRISMA Flow Diagram

Table S1. Search strategies

MEDLINE via PubMed	("Ostomy"[Mesh] OR "Colostomy"[Mesh] OR "Ileostomy"[Mesh] OR "Urostomy"[Mesh] OR ostom*[tiab] OR stom*[tiab] OR colostom*[tiab] OR ileostom*[tiab] OR urostom*[tiab] OR enterostom*[tiab] OR ureterostom*[tiab] OR "ureteroileocutaneostomy"[tiab] OR "ileal conduit"[tiab] OR "urinary diversion"[tiab]) AND ("Patient Education as Topic"[Mesh] OR "Health Education"[Mesh] OR educat*[tiab]) AND ("Telemedicine"[Mesh] OR "Remote Consultation"[Mesh] OR "Distance Education"[Mesh] OR telehealth[tiab] OR telemedicine[tiab] OR telenursing[tiab] OR ehealth[tiab] OR e-health[tiab] OR "remote education"[tiab] OR "distance education"[tiab] OR online[tiab] OR web-based[tiab] OR internet-based[tiab] OR "mobile application*"[tiab])	208
CINAHL	((MH Ostomy+)OR (MH Colostomy+)OR (MH Ileostomy+)OR (MH Urostomy+)OR (TI ostom* OR AB ostom*)OR (TI stom* OR AB stom*)OR (TI colostom* OR AB colostom*)OR (TI ileostom* OR AB ileostom*)OR (TI urostom* OR AB urostom*)OR (TI enterostom* OR AB enterostom*)OR (TI ureterostom* OR AB ureterostom*)OR (TI ureteroileocutaneostomy OR AB ureteroileocutaneostomy)OR (TI "ileal conduit" OR AB "ileal conduit")OR (TI "urinary diversion" OR AB "urinary diversion"))AND((MH "Patient Education as Topic+")OR (MH "Health Education+")OR (TI educat* OR AB educat*))AND((MH Telemedicine+)OR (MH "Remote Consultation+")OR (MH "Distance Education+")OR (TI telehealth OR AB telehealth)OR (TI telemedicine OR AB telemedicine)OR (TI telenursing OR AB telenursing)OR (TI ehealth OR AB ehealth)OR (TI e-health OR AB e-health)OR (TI "remote education" OR AB "remote education")OR (TI "distance education" OR AB "distance education")OR (TI online OR AB online)OR (TI web-based OR AB web-based)OR (TI internet-based OR AB internet-based)OR (TI "mobile application*" OR AB "mobile application*"))	140
Scopus	TITLE-ABS-KEY ((Ostomy OR Colostomy OR Ileostomy OR Urostomy OR Enterostomy OR "ostom" OR "stom" OR "colostom" OR "ileostom" OR "urostom" OR "enterostom" OR "ureterostom" OR ureteroileocutaneostomy OR uretero-ileocutaneostomy OR "urinary diversion" OR "intestinal stoma" OR "fecal stoma" OR "faecal stoma" OR "urinary stoma") AND ("Patient Education" OR "Health Education" OR "educat" OR "instruct" OR "patient education" OR "health education") AND ("Remote Consultation" OR "Distance Education" OR "telehealth" OR "telecare" OR "remote education" OR "remote intervention" OR "remote consultation" OR "remote care" OR "distance education" OR "distance learning" OR "online" OR "web-based" OR "internet-based" OR "videoconferenc" OR "virtual care" OR "virtual visit" OR "non-face-to-face"))	40
Web of Science	(Ostomy OR Colostomy OR Ileostomy OR Urostomy OR ostom* OR stom* OR colostom* OR ileostom* OR urostom* OR enterostom* OR ureterostom* OR ureteroileocutaneostomy OR "ileal conduit" OR "urinary diversion") AND ("Patient Education as Topic" OR "Health Education" OR educat*) AND (Telemedicine OR "Remote Consultation" OR "Distance Education" OR telehealth OR telemedicine OR telenursing OR ehealth OR e-health OR "remote education" OR "distance education" OR online OR web-based OR internet-based OR "mobile application*")	183

Remote Patient Education for People Living with an Ostomy: An Italian Expert Consensus Using a Modified Delphi Method

Figure S1. PRISMA Flow Diagram
Here below, we report the PRISMA 2020 flow diagram describing the literature search conducted to develop the preliminary consensus statements.

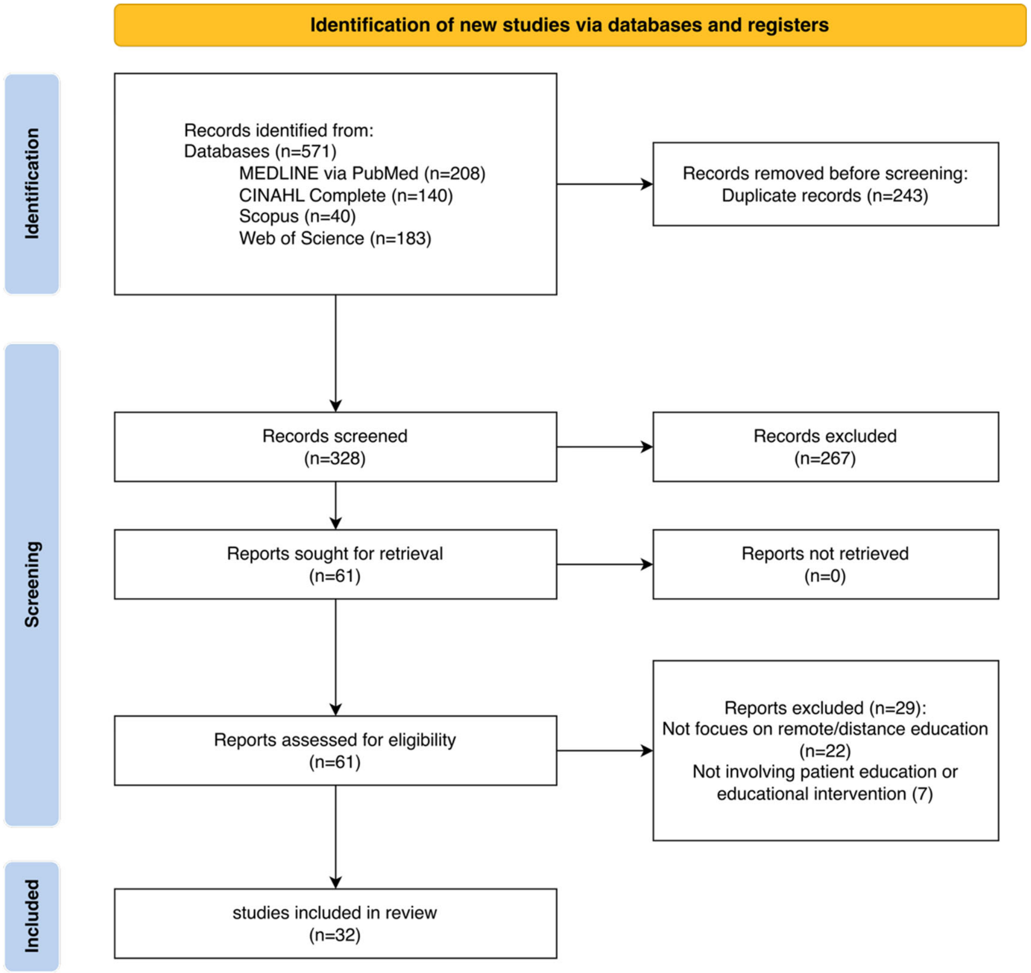

Remote Patient Education for People Living with an Ostomy: An Italian Expert Consensus Using a Modified Delphi Method

Risk of bias assessment

Table S2. Quantitative studies (MMAT)

Paper	Selection / allocation	Measurement (validity/objectivity)	Confounding / comparability	Missing data / non-response	Analysis	Overall
**Coffey et al., 2022**	🔴	🟠	🔴	⚪	🟠	🔴
**Gusdorf et al., 2023**	🟠	🟢	🟠	⚪	🟢	🟠
**Kristosakis et al., 2021**	🟠	🟠	🟠	⚪	🟢	🟠
**Schröder et al., 2023**	🔴	🟠	🟠	🔴	🟠	🔴
**Si et al., 2022**	🟢	🟠	🟢	🟠	🟢	🟠

Table S3. Qualitative studies (CASP qualitative)

Paper	Appropriate design	Recruitment / sample	Data collection	Reflexivity (researcher–participant relationship)	Analysis rigour	Credibility of findings	Overall
**Kim et al., 2024**	🟢	🟠	🟢	⚪	🟢	🟢	🟠
**Mash et al., 2023**	🟢	🟠	🟢	🟠	🟢	🟢	🟠
**Soares-Pinto et al., 2023**	🟢	🔴	🟠	⚪	🟠	🟠	🔴

Table S4. Reviews (ROBIS)

Paper	D1 Eligibility criteria	D2 Identification & selection	D3 Data collection & appraisal	D4 Synthesis & findings	Overall
**Gordon et al., 2023** (Cochrane Systematic Review)	🟢	🟢	🟢	🟠	🟢
**Guirado-Fuentes et al., 2023** (Scoping review)	🟠	🔴	🔴	🔴	🔴
**Martínez-Miranda et al., 2023** (Systematic Review and Network Meta-Analysis)	🟢	🟠	🟠	🟠	🟠

Table S5. Paper not assessable

Paper	Category	Result
**Hamid et al., 2023**	Education/programme description	(N/A)
